# CcpNmr AnalysisAssign: a flexible platform for integrated NMR analysis

**DOI:** 10.1007/s10858-016-0060-y

**Published:** 2016-09-23

**Authors:** Simon P. Skinner, Rasmus H. Fogh, Wayne Boucher, Timothy J. Ragan, Luca G. Mureddu, Geerten W. Vuister

**Affiliations:** 1 Department of Molecular and Cell Biology, Leicester Institute for Structural- and Chemical Biology, University of Leicester, Leicester, UK; 2 Department of Biochemistry, University of Cambridge, Cambridge, UK

**Keywords:** NMR, Data analysis, Software, CCPN, Python, Assignment

## Abstract

NMR spectroscopy is an indispensably powerful technique for the analysis of biomolecules under ambient conditions, both for structural- and functional studies. However, in practice the complexity of the technique has often frustrated its application by non-specialists. In this paper, we present CcpNmr version-3, the latest software release from the Collaborative Computational Project for NMR, for all aspects of NMR data analysis, including liquid- and solid-state NMR data. This software has been designed to be simple, functional and flexible, and aims to ensure that routine tasks can be performed in a straightforward manner. We have designed the software according to modern software engineering principles and leveraged the capabilities of modern graphics libraries to simplify a variety of data analysis tasks. We describe the process of backbone assignment as an example of the flexibility and simplicity of implementing workflows, as well as the toolkit used to create the necessary graphics for this workflow. The package can be downloaded from www.ccpn.ac.uk/v3-software/downloads and is freely available to all non-profit organisations.

## Introduction

NMR spectroscopy is an incredibly powerful, non-invasive, analytical technique used in many areas of research, including materials science, medical diagnosis, industrial process control and chemistry (ur-Rahman and Choudhary 2015). It is also used in many fields of biomolecular research, including structural biology, enzymology, signal transduction, physiology and drug discovery (Bertini et al. 2012). NMR enables the study of systems at the atomic level under conditions similar to those in cellular systems, and even in cells themselves (Ikeya et al. 2010). NMR observables such as chemical shifts, peak intensities, scalar and dipolar couplings, line widths, and cross-relaxation rates provide critical inter and intra-molecular information about the molecule(s) under study (Vuister et al. 2011). One major advantage of NMR in the study of biological systems is its ability to detect local changes in conformation and dynamics, otherwise undetected by other techniques that play functional roles (Baldwin and Kay 2009; Anthis and Clore 2015).

At present there is a range of software solutions for handling and analysing various multidimensional NMR data sets, and their derived data, such as restraints, or rates, as well as the associated bookkeeping. According to BMRB statistics, the five most cited packages for NMR analysis are Sparky (Goddard and Kneller), Cara (Keller 2005), CcpNmr Analysis (Vranken et al. 2005), NmrView (Johnson and Blevins 1994) and XEASY (Bartels et al. 1995), although such analysis is somewhat flawed as the depositors are not required to specify the software used for their studies. This is further highlighted by the fact that only 70 % of all BMRB entries explicitly specify the software used. Each of these NMR software packages has its own interpretation of and resultant workflows for NMR analysis, although at their core, the basic principles used to interpret the NMR data are very similar. As working with a particular NMR software package involves a sometimes steep learning curve, a user will tend to stick with the package that they have used previously and hence are familiar with. Additionally, exchange of data between NMR software packages has proven to be cumbersome, and thus complicated the application of data analysis routines not available within the package of choice. The emerging NMR Exchange Format (NEF) is designed to alleviate this situation (Gutmanas et al. 2015), and the new version-3 software is fully NEF compatible (vide infra).

Since its inception, the CCPN has sought to overcome the difficulties mentioned above as well as developing software for analysing NMR data for a variety of different purposes and across all fields in the NMR community. The flagship package of the CCPN, namely, CcpNmr Analysis (Vranken et al. 2005), released in 2003, is now at version 2.4 and its user community continues to grow year on year. CcpNmr Analysis was inspired by the interface and peak-picking routines of the program Sparky, and by the spectrum display and spin-system handling of the program ANSIG. Its development was aimed to alleviate some of the limitations of these two programs whilst combining their desirable aspects. Analysis was built on top of the CCPN data model (Fogh et al. 2010) and its data access libraries, which determined the program’s philosophy. This exact modelling of all the data contained within the CCPN project ensures that its persistence is guaranteed and its interpretation is precisely defined. CcpNmr Analysis was designed to accommodate any desired assignment strategy and to allow full access to all internal data using a public, and documented API; thus facilitating the writing of macros, or code, to extend the functionality of the program. However, the number of user-written extensions has thus far been limited. Anecdotal evidence suggests that this is, in part, due to the complexity of the data model and the deep level of understanding of the code base required to write a customised extension.

In this paper, we present the latest version of the CcpNmr software, namely, CcpNmr version-3, which represents a complete overhaul of the existing package. CcpNmr version-3 ultimately will consist of a suite of programs, developed around a unified code base, with dedicated functions such as assignment, NMR structure calculations, NMR-based screening or NMR-based metabolomics. Here, we present CcpNmr version-3 AnalysisAssign, subsequently denoted as AnalysisAssign, which specifically targets the inspection, analysis and assignment of NMR spectral data.

During the design phase of AnalysisAssign, we queried both our own and users of other programs as to the requirements and work practices that a modern-day NMR data analysis package should support. AnalysisAssign has been written using modern software engineering principles, including the complete and clean separation of graphical and data code. This separation ensures that graphical developers need only interact with graphical code and do not need a deep understanding of data handling routines and vice versa. Hereby, data routines and graphical interfaces can be developed independently and then fused together when needed. This also ensures a greater level of maintainability and testability of the code base, leading to highly robust and stable software.

On the graphical side, we have chosen PyQt4 as the graphical engine. PyQt4 is both cross-platform and native to any operating system; the latter has enabled us to leverage the capabilities of individual platforms, whilst also being able to develop a bespoke and highly functional graphical interface. We have removed what has been described as “the popup explosion”, characteristic of CcpNmr Analysis version 2.4, and replaced it with a self-contained, single window interface (vide infra, Fig. 2), creating a cleaner user interface and user experience for facile navigation of the software. We have incorporated drag-and-drop for loading of all compatible data types and as the basis of some workflows within the software, ensuring that simple tasks are simple to perform.

To facilitate the facile construction of software extensions, we have taken PyQt4 as a base and built a toolkit of widgets that can be assembled into fully functional graphical units in a straightforward manner. Moreover, we have created a so-called “wrapper layer” around the main CCPN data API (Fogh et al. 2010) to enable access to the data via a simple, Python-based, command interface. Using this wrapper layer, a user can create their own macros in a language that spectroscopists understand. This transparency of both graphical components and data access make the CcpNmr version-3 software platform very easy to extend without having to learn an entire software framework first, as is currently the case with Sparky, for example.

## Methods

AnalysisAssign was written in version 3.5 of the Python programming language. The PyQt4 and PyQtGraph 0.9.10 (www.pyqtgraph.org) graphical libraries were used to construct the graphical user interface (GUI) and data displays. The NumPy 1.11.1 (van der Walt et al. 2011), SciPy 0.17.1 (Jones et al. 2001), Pandas 0.18.0 (McKinney 2010) and Sci-kit learn 0.17.1 (Pedregosa et al. 2011) Python libraries were used for the construction of algorithms and workflows. Code for contouring, peak picking and peak fitting was written in the C programming language and an SVN repository, hosted by Sourceforge (www.sourceforge.net), was used for version control.

Distributions of AnalysisAssign are available for Mac OSX, a number of Linux environments and as a VirtualBox image from www.ccpn.ac.uk/v3-software/downloads; all of which include an Anaconda Python distribution (Continuum Analytics, TX, USA) and work without the need for setup or compilation of code. Moreover, we have put tutorial projects, instructions, example data and the complete program-specific documentation inside all distributions of the software, in order to provide the users with ample resources to start working with the program.

PyQt has a wide range of different widgets for different tasks. The syntax to connect different widgets to each other and assemble them into higher order structures varies depending on the type of widget and its purpose. We denote the top-level GUI elements as modules, each intended to either represent the data and/or to allow interaction with the data. There are modules for spectral display, peak tables, sequence, notes, console, etc., which can be combined freely to construct a functional program, and these will be discussed in detail below. We have implemented a uniform syntax for all widgets and modules used in the software to make the creation of modules more straightforward. Furthermore, the assembly of widgets within the modules is performed using a gridding system, which facilitates rapid development of even complex modules. In PyQt, widgets can be assembled into higher-order layouts using absolute or relative positioning (akin to CSS3). The gridding system used in CcpNmr version-3 uses a modified version of PyQt relative positioning, such that the position of any given widget can be specified in a straightforward fashion by two grid coordinates and an optional grid span. Using these two parameters, widgets can be very easily arranged inside modules. All modules inside AnalysisAssign have been built using this toolkit, and thus serve as examples and templates for users to construct their own bespoke modules for specific tasks, or even to build upon existing modules and extend them.

### The data

**Fig. 1:**
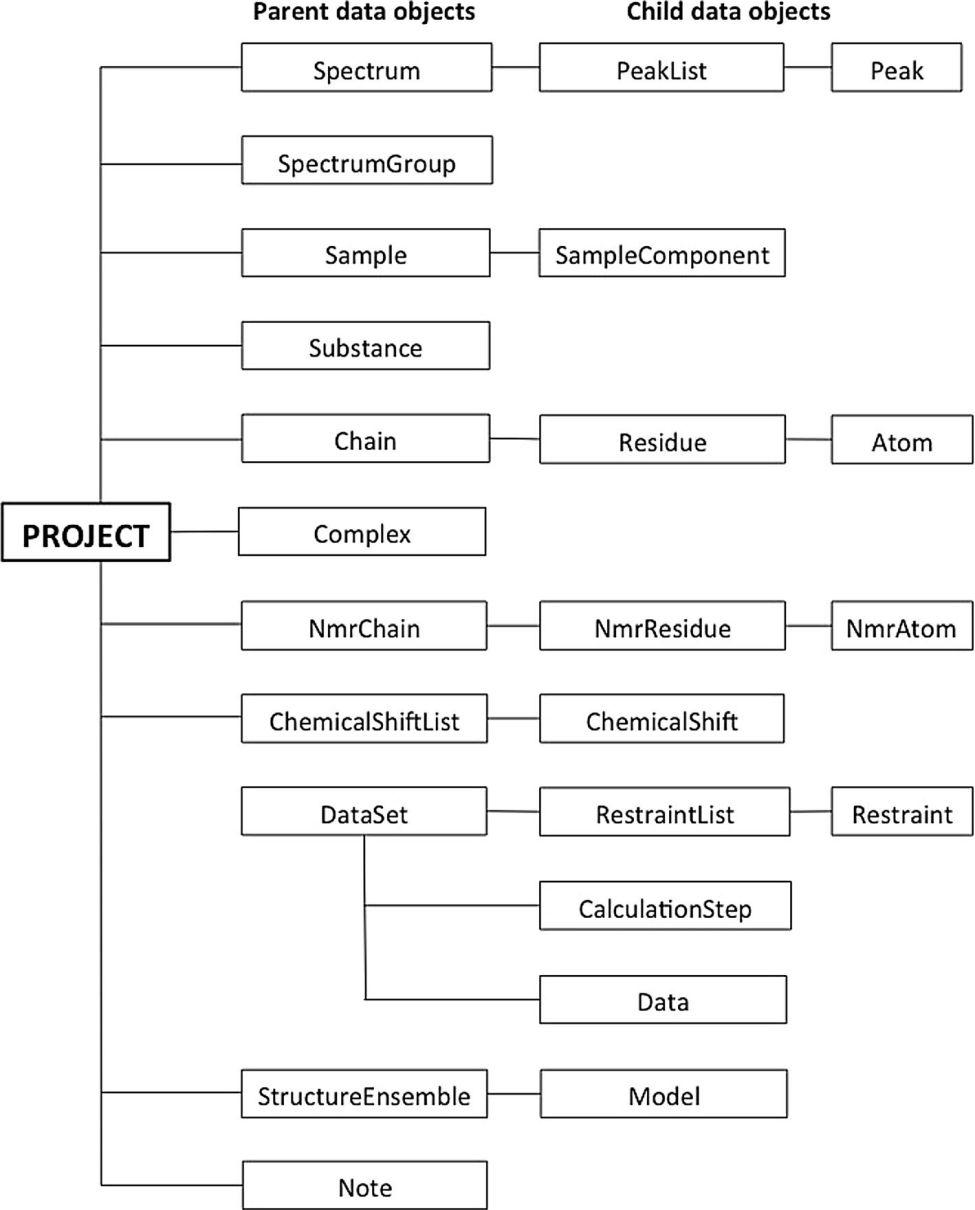
Overview of the hierarchical data structure of an AnalysisAssign project. The data objects cover all data that can be stored in a project. A Spectrum is an object containing the path to the stored NMR data file and all the stored properties of that data. A PeakList is a child object of a Spectrum and serves as a container for Peak objects, which contain all position, intensity and assignment information. A SpectrumGroup combines multiple Spectrum objects, so they can be treated as a single object. A Sample corresponds to an NMR sample and is made up of SampleComponents, which indicate the individual chemical entities that make up that Sample, e.g. protein, buffer, salt, and their concentrations. A Substance object represents a defined chemical entity. A Chain object corresponds to a molecular chain, made up of Residues, which in turn are made up of Atom objects. A Complex is a container for multiple chain objects. NmrAtom objects serve as a way of connecting a named nucleus to an observed chemical shift; peaks are assigned to NmrAtoms. NmrAtoms are grouped into NmrResidues, which in turn are grouped into NmrChains. A ChemicalShiftList object is a container for ChemicalShift objects, which represent observed chemical shifts. DataSet objects serve to group RestraintLists and other input and output from a calculation, with the Data object storing links to the data structures used (PeakLists, Spectra, StructureEnsembles etc.) and associated calculation parameters. A RestraintList contains Restraint objects of a specific type (distance, dihedral, etc.). Restraint objects contain the measured value of that restraint, information for force field calculations, and the Atoms involved in the restraint. CalculationStep objects are used to track the calculation history of a DataSet, with the programs used for previous steps and their input and output DataSets. A StructureEnsemble object is a container for ensembles of coordinate structures, with each coordinate structure defined by a Model object. Notes are objects that contain free-text information to be stored in a project

NMR data in AnalysisAssign projects are organised in a hierarchical structure referred to as a Project (Fig. 1). There are eleven top-level, parent data objects in a project, of which seven have child objects, namely, Spectrum, Sample, Chain, NmrChain, ChemicalShiftList, DataSet and StructureEnsemble. The child data objects are organised in a logical hierarchy; for example, a Spectrum has PeakLists, which in turn, are made up of Peaks, whereas a Chain is made up of Residues, which are made up of Atoms. This data hierarchy is presented to the user in the sidebar of the graphical user interface (GUI; vide infra) and provides a clean way for users to navigate the multitude of different data objects that a project may contain. Within a project, all individual data objects have a so-called “project ID” or PID. The PID is a unique, systematically constructed string corresponding to a specific piece of data, for example a peak, a spectrum or an atom, and reflecting its hierarchical relationship, very much like a URL. In this way, any piece of data can be accessed from anywhere within the software in a straightforward way.

The NmrChain object and its descendent objects, NmrResidues and NmrAtoms are central to the assignment philosophy of AnalysisAssign and thus will be explained here. An NmrAtom serves as a way of connecting a named nucleus to an observed chemical shift; peaks are assigned to NmrAtoms. These NmrAtoms are grouped into NmrResidues, which in turn are grouped into NmrChains. This is a parallel hierarchy to Chains, Residues and Atoms, which represent the component parts of the molecule(s) under study. The NmrChain, NmrResidue and NmrAtom objects can be given any name desired; however, some rules and functional logic convey order to this process. The flexibility of naming, in particular NmrResidues, is incredibly useful in the different steps of protein assignment. NmrResidues can be created and just be given an index; e.g. @13, and leaving any residue type description either undefined or ambiguous, if so desired. However, it is also possible to name a residue with an offset relative to another NmrResidue, e.g. an NmrResidue called @13-1 would be the NmrResidue at a -1 offset to NmrResidue @13. This concept is used heavily in the implemented assignment procedures of AnalysisAssign.

In contrast to the NMR-related objects, the names of the molecular Chain, Residue and Atom objects are much more restricted and, in fact, directly follow the NEF syntax specification (Gutmanas et al. 2015). Crucially, NmrChain, NmrResidue and NmrAtom objects are deemed “assigned” when their names match the corresponding molecular objects in the parallel hierarchy, i.e. if the name of an NmrResidue matches the name of a Residue, this NmrResidue is considered assigned to that Residue and a dynamic connection between the two objects is established. By using this setup, we have secured flexibility in the assignment process, while simultaneously assuring that the end result would comply with valid molecular descriptors.

AnalysisAssign is fully compatible with CcpNmr version-2 projects (Vranken et al. 2005), and an on-the-fly conversion of the version-2 project preserves all data and metadata. The program is also capable of reading all major NMR spectral data formats natively, including Bruker (Bruker Biospin, Germany), Varian/Agilent (Agilent Technologies, USA), NmrPipe (Delaglio et al. 1995) and UCSF formats. By default, the NMR spectral data are not contained within the project. Instead, references to the data are maintained. A simple mechanism specifying a common location for these spectral data has been implemented in AnalysisAssign, allowing for a straightforward way to specify relative locations for the individual spectra. AnalysisAssign can read Fasta, PDB-v3 and NEF (Gutmanas et al. 2015) files for defining molecular sequences with more formats, e.g. mmCIF, to follow. For an overview of the different file formats, see Table 1.

**Table 1:** Different data formats that can be loaded and parsed by AnalysisAssign

Format	Drop on sidebar	Load via menu	Drop onto module	Action
CCPN version 2 project	✓	✓	✓	convert to version 3 and load project
CCPN version 3 project	✓	✓	✓	load project
NmrPipe	✓	✓	✓	loads and displays spectrum^a^
Azara	✓	✓	✓	loads and displays spectrum^a^
Bruker	✓	✓	✓	loads and displays spectrum^a^
Varian/Agilent	✓	✓	✓	loads and displays spectrum^a^
NmrView	✓	✓	✓	loads and displays spectrum^a^
XEASY	✓	✓	✓	loads and displays spectrum^a^
UCSF	✓	✓	✓	loads and displays spectrum^a^
FASTA	✓	✓		create molecule(s) from sequence
PDB-v3	✓	✓		load structure(s)
NEF	✓	✓		parse contents and load into project
comma separated file	✓	✓		parse contents and load if possible
Excel spread sheet	✓	✓		parse contents and load if possible

^a^When dropped on spectrum display module

### The graphical user interface

**Fig. 2:**
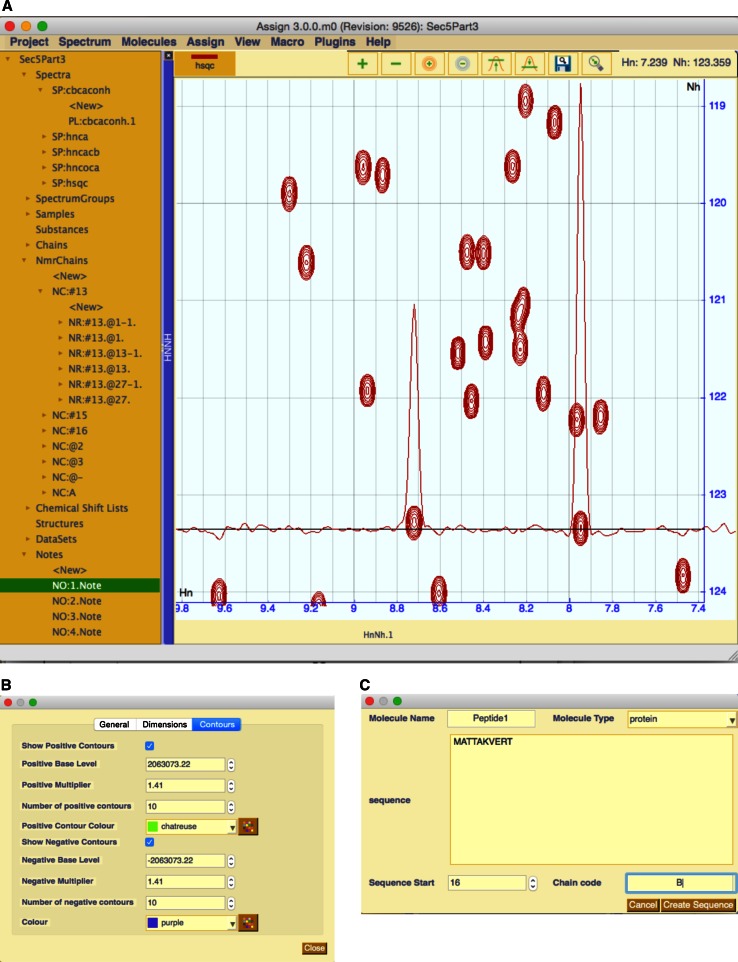
**a** The main GUI of AnalysisAssign consists of four main areas: the sidebar (*left*), the menubar (*top*), the status bar (*bottom*) and the module display area. The sidebar contains links to all data in the project and many of the headings several objects underneath them. The <New> items underneath the objects enable simple creation a child data objects. The module display area contains a spectrum display module (cf. Table 2) for a two-dimension spectrum with a horizontal trace in the Hn dimension. The trace colour is the same as the positive contour colour by default. **b** A dedicated popup for changing spectrum properties, which contains three tabs for different types of properties. The selected tab provides facilities to adjust the display properties of this spectrum. **c** The dedicated popup for chain creation. This appears when the <New> button under chains is activated

The main graphical user interface (GUI) of AnalysisAssign is separated into four areas—the menu bar, the sidebar, the status bar and the module display area (Fig. 2a). The menu bar provides access to a variety of actions for loading, displaying and interacting with data. Many actions in AnalysisAssign also have two-key shortcuts, to allow for their rapid execution. For actions accessible from the menu bar, their corresponding two-key shortcuts are specified alongside the corresponding menu item. A list of all shortcuts is also accessible from the Help menu.

The sidebar (Fig. 2a) contains links to all of the data contained in a project and it follows the hierarchical data structure of a project (described in the data section, cf. Fig. 1). Data can be loaded into the project either via the menus, or by using drag-and-drop onto the appropriate areas (cf. Table 2). The program automatically detects the file format and will create the appropriate data objects for display in the sidebar.

**Table 2:** List of modules currently implemented in CcpNmr version-3 and their associated functionalities

Module (shortcut)	Functionality	Adjustable parameters
Backbone Assignment Module (bb)	Ordering strips for backbone assignment	✓
Pick And Assign Module (pa)	Facilitate pick and assign workflow.	✓
Sequence Graph (sg)	Display assignment information	
Peak Table (lt)	Display peak lists	
Chemical Shift List (ct)	Display chemical shift lists	
NmrResidue Table (nt)	Display NmrResidue information	
Reference Chemical Shifts (rc)	Display reference chemical shift ranges for protein residues.	
Peak Assigner (aa)	Assign NmrAtoms individual peak dimensions	
Atom Selector (as)	Assign NmrAtoms to peaks.	
Spectrum Display (nd)	Display compatible spectra.	
Ipython console (py)	Ipython interface to the application.	
Sequence display (sq)	Displays all molecules inside the project.	

Some modules have adjustable parameters as indicated

Double clicking data objects in the side bar will raise a popup that allows the user to change the properties of that particular data object. As an example, Fig. 2b shows the popup for a spectrum. Using this popup, a user can change a multitude of different properties directly associated with this particular spectrum, such as its contour colours, numbers of contour levels, name and assignment tolerances, to name a few. A user can also view information related to the different dimensions of the spectrum as well as other more general properties such as the spectrum name and data path.

Whenever appropriate, new data objects can be created interactively using the sidebar by double clicking ‘<New>’ underneath the parent data object, e.g. a new empty peak list can be created for a specific spectrum using the <New> underneath that spectrum (Fig. 2a). Its properties can be subsequently modified as well. In some circumstances, the <New> operation will initiate a dedicated popup that populates the object with relevant information/data. An example of this is the New Chain popup (Fig. 2c), which allows for the straightforward definition of a new Chain and its Residue objects. Objects can also be deleted by a context-dependent right mouse click.

**Fig. 3:**
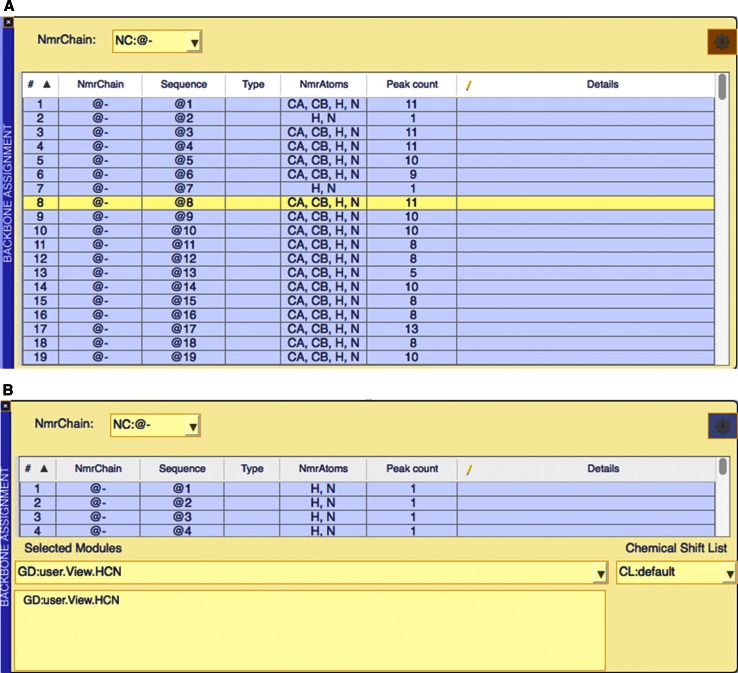
**a** The Backbone Assignment module. This module contains an NmrResidue table to which spectral navigation functionality has been attached. **b** The Backbone Assignment module’s ‘gear’ button reveals a second layout containing the adjustable parameters relevant to the backbone assignment workflow

Inside the module display area, a variety of different so-called “modules” can be displayed (Table 2). A module is a functional graphical unit designed for a specific task e.g. spectrum display and interaction or data display/manipulation. Modules can be re-arranged into any configuration, independently resized and also ‘popped out’ of the module display area into an independently displayed graphics window, giving the user a very flexible way to display and arrange the various modules. Once a module is outside the module display area it behaves as a separate window, in the operating system sense. It can also be put back into the module area via drag-and-drop. These modules are assembled using a toolkit of widgets, e.g. labels, textboxes, drop down lists, which we have developed for facile GUI programming in the context of NMR spectroscopy. Moreover, each module consists of two layouts, the main layout, which is always visible, and a settings layout that can be shown and hidden (Fig. 3b) via a nowadays familiar ‘gearbox’ button. In the settings layout any adjustable parameters required for a specific task contained by the module can be set and changed.

### Spectrum display modules

**Fig. 4:**
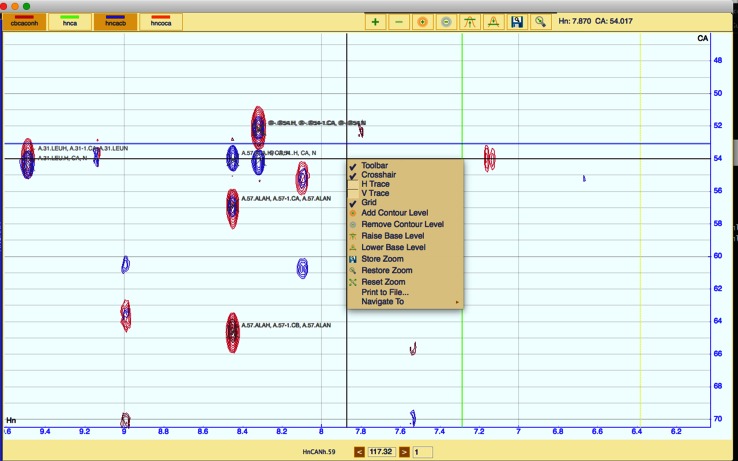
A Spectrum display module containing four 3D triple-resonance spectra, i.e. cbcaconh, hnca, hncacb and hncoca. The spectrum buttons have darker background when displayed to indicate that the button has been pressed and consequently the spectrum is visible. Peaks, displayed as crosses, together with assignment annotations are also displayed. The context menu contains many of the tools shown in the toolbar along with some additional tools. The navigation bar at the bottom of the module enables scrolling through the third dimension of the spectrum

Spectrum display modules (Fig. 4) can contain any number of spectra as long as the axes of all the spectra are compatible with each other. This compatibility is determined by the nature of the experiment, i.e. the number of dimensions, nuclei observed. AnalysisAssign automatically determines a so-called “axisCode”, e.g. H, N, C, etc., of each dimension of the experiment upon reading the spectral data the first time. The program also has over 300 pre-defined experiment types available to the user along with the ability to add any other experiment type a user may require. Selecting such a predefined type defines the axisCodes more explicitly; e.g. the two dimensions of the [^15^N,^1^H]-HSQC will be set to Hn and Nh, denoting the two covalently attached nuclei. For two spectra to be displayed in the same spectrum display module, their axis codes must be compatible, i.e. a [^15^N,^1^H]-HSQC spectrum cannot be displayed in the same module as a [^13^C,^1^H]-HSQC spectrum.

The experiment types are also used to determine which atoms are observable in a given experiment and facilitate the prediction of assignment possibilities and expected peak patterns. To use many of the tools for assignment in AnalysisAssign, the proper experiment type has to be specified, which can be done via either the spectrum properties or via a dedicated popup, activated from the Spectrum menu or by using the ‘et’ shortcut command.

The spectrum display module consists of three main areas: the toolbar, the spectrum widget, which can simultaneously display an arbitrary number of spectra, and the navigation area. On the left hand side, the toolbar contains the spectrum buttons to toggle the display of individual spectra or peak lists. The middle region contains buttons to manipulate the displayed spectra for easy access to frequently used functionalities; its display can be switched on and off (shortcut ‘tb’). The right hand side shows the current position of the mouse inside the spectrum widget. All spectrum display modules are in-fact so-called “strip displays”. Strips convey multiple views into the same spectrum, which is particularly convenient when inspecting 3D or 4D spectra. When a spectrum display module is first created, it contains only one strip and additional strips can be added using the toolbar. The axes of each strip can be zoomed concurrently and independently depending on the location of the mouse pointer, hereby, the aspect ratio of the strip can be dynamically modified.

A context menu can be accessed in each strip, which contains many of the functions available in the toolbar along with some additional tools, e.g. printing the current display and showing/hiding the gridlines.

The navigation area of a spectrum display module will differ according to the dimensionality of the spectra displayed. If 1D or 2D spectra are displayed, the navigation area will only contain a strip PID and, in some cases, an NmrResidue PID. If spectra with three or more dimensions are displayed, the navigation area will also contain a set of tools for navigating through the additional dimensions (planes) of the spectra, either by stepping through planes using the arrow buttons or entering a specific chemical shift position into the text box. The depth of the displayed planes of the additional dimensions can also be set so that a user can display any number of planes concurrently.

### Functional tables

All table modules (e.g. Fig. 3a) in AnalysisAssign are highly functional units and serve to both enable display and sorting of different types of tabular data, such as peak lists, chemical shifts lists or NmrResidue lists, and optionally have specific functionalities attached to them. In this way, specific analysis routines can be controlled from the selection of specific data in the table. For example, routines for backbone and side chain assignment of proteins are both driven from tables of NmrResidues. These tables have been connected to spectrum display modules and to functions that handle spectrum navigation and assignment. The functionalities attached to the NmrResidue table for backbone assignment will be discussed in more detail later in this paper. The ability to attach functionality to tables of data enables the construction of simple interfaces for a broad range of data analysis tasks. Currently, additional modules for backbone and side chain assignment of protein nuclei and plotting data exist, and new ones are under continuous development.

### Support and extensions

In AnalysisAssign, we have created various facilities to support users of the software and those seeking to create their own extensions to the software via so-called “plugins”.

In the Help menu, we have links to all published tutorial instructions, manuals and program-specific documentation, which are contained within the installation. The manuals and documentation contain all the information required to produce anything from task specific macros to fully-fledged application plugins. In order to allow easy integration of external software, we have facilities to handle plugin code seamlessly and launch it from within the application. We have also implemented a facility to submit macros to a repository; any macros that are considered useful additions to the program will be included in subsequent distributions.

### The IPython console

The IPython console is one of simplest, yet most powerful modules inside AnalysisAssign. It is simple in a graphical sense, since it only contains four widgets, however, the underlying functionality built into this module enables complete control and navigation of both the data and graphics using simple, Python-based commands. Inside the IPython console is a fully functional IPython command line interpreter (Pérez and Granger 2007) and therefore native Python code can be written and executed inside it. Moreover, the IPython namespace has been extended to include “project”, which corresponds to the current project loaded into the software; “application”, which provides access to a wide variety of application level functions, for example, displaying a peak table; “current”, which contains pointers to currently selected items;  and “mainWindow”, which provides access to lower-level graphical functionality. The combination of the namespace extension and the data wrapper forms a simple macro language, whereby otherwise complex or repetitive tasks can be automated in a few short commands. In addition to the IPython command line, the IPython console contains a so-called “echo display.” This echo display contains the programmatic version of all actions performed in the GUI, such that all echoed commands could be put into a macro and the exact states of the data and GUI up to that point could be faithfully reproduced.

## Case study: backbone assignment

One of the first steps of an NMR study is the assignment of the protein of interest. There are various manual, semi-automated and automated algorithms (Bartels et al. 1997; Zimmerman et al. 1997; Grishaev and Llinas 2002; Jung and Zweckstetter 2004; Vitek et al. 2005; Xu et al. 2005; López-Méndez and Güntert 2006; Bahrami et al. 2009) implemented within different software packages to perform assignment. In AnalysisAssign, we have taken many of the core components that constitute these routines and assembled them into two complete workflows for backbone and side-chain NMR assignment. We have also implemented inherent flexibility and modularity in the design of these workflows, such that they can be easily customised and adapted to specific needs.

The first step in any assignment procedure in AnalysisAssign is the grouping of related NmrAtoms into NmrResidues, which can then be assigned to Residue objects once their location in the macromolecular sequence has been determined. This process is performed using the “pick-and-assign” workflow. To initiate this process, a peak is picked in a reference spectrum, e.g. a [^15^N,^1^H] HSQC or HNCO spectrum, from which NmrAtoms, and by virtue, NmrResidues, are created using the peak positions. Subsequently, the peak positions are used to pick peaks in related spectra, e.g. triple-resonance HNCACB and CBCAcoNH spectra, and assign the NmrAtoms to these peaks along the corresponding dimensions. Finally, the unassigned dimensions of these peaks are used to create additional NmrAtoms.

**Fig. 5:**
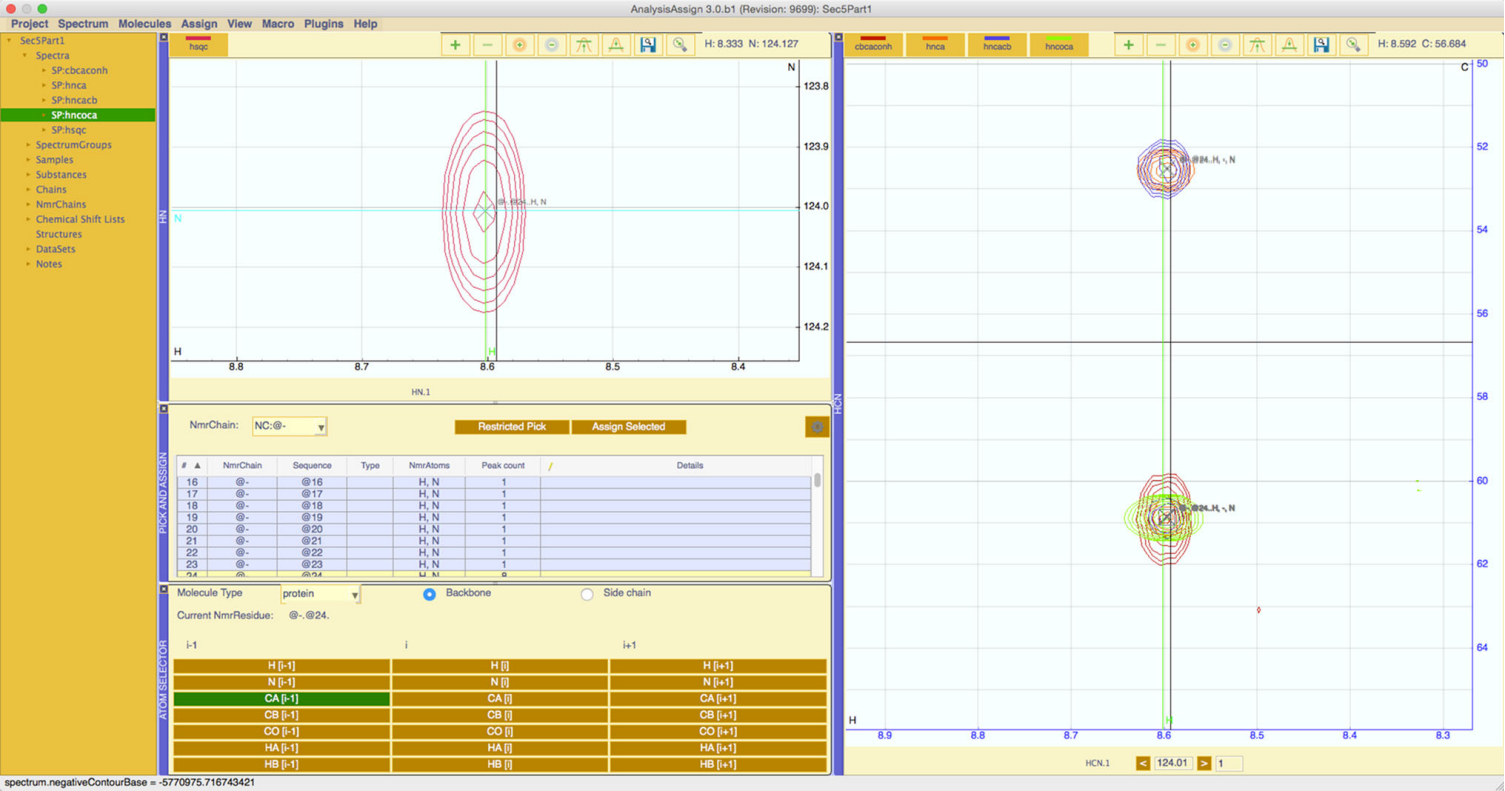
A layout for carrying the pick and assign workflow. The peaks in the 3D spectra have been assigned to the selected NmrResidue. The atom selector shows a prediction that the selected peaks all correspond to a CA^*i*−*1*^ NmrAtom. The spectrum display modules contain labelled markers at the ^1^H and ^15^N chemical shift positions

This procedure is called the “pick-and-assign” workflow inside AnalysisAssign and this workflow is controlled principally from the Pick and Assign module (Fig. 5). This module contains an NmrResidue table with spectral navigation functionality built into it. When an NmrResidue is activated in the table by double-clicking a specific row, all spectrum display modules navigate to the positions defined by the chemical shifts of the NmrAtoms that make up the NmrResidue, and these positions are marked on the corresponding displays. This module also contains the facilities to perform a restricted peak pick along y-axes of the spectrum display modules, where the x and z ranges are restricted by the chemical shift values of the NmrAtoms in the NmrResidue and can be set accordingly. The remaining dimension(s) of the newly picked peaks can then be assigned to relevant NmrAtom(s) using the Atom Selector module, which implements selection logic based upon the possible transfers derived from the experiment type.

The Atom Selector module (displayed in Fig. 5) has been designed to work in concert with the pick and assign module to easily assign NmrAtoms to selected peaks. In the backbone assignment case, selecting an NmrResidue inside the pick and assign module and then selecting a group of peaks that overlay will cause the atom selector will predict whether an unassigned peak dimension corresponds to CA, HA, CB or HB. The atom selector will also indicate if the peak dimension corresponds to an intra-residue or an inter-residue atom, based on the experiment type. The combination of these two modules in the pick and assign workflow make the process of setting up a backbone assignment very straightforward.

**Fig. 6:**
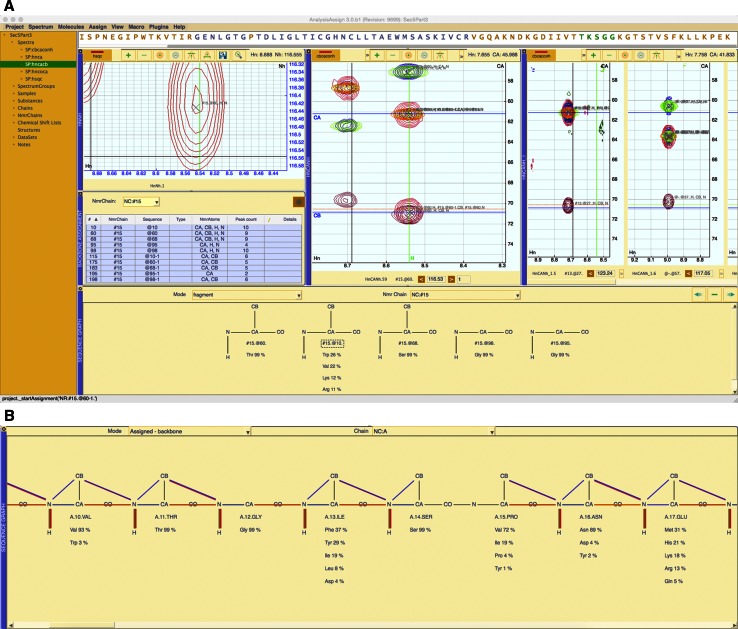
**a** Layout of the modules in AnalysisAssign for backbone assignment. Two of the spectrum display modules are query and match modules for the ordering of strips and the third spectrum display module shows a Ref. [^15^N, ^1^H] HSQC spectrum. The backbone assignment module drives this workflow; the Sequence Graph module shows the NmrResidues built into a connected stretch with corresponding residue type predictions and the Sequence Module at the top shows a prediction of the sequence location of the connected stretch. **b** Once NmrResidues have been assigned, the Sequence Graph can be used to display from which spectrum or spectra these assignment come. Connecting lines in the display are derived from the assigned peaks in the different spectra and displayed in the appropriate positive contour colour of that spectrum

The default backbone assignment workflow developed for AnalysisAssign is currently based on, however *not inherently limited* to, linking strips via inter- and intra-residual CA and CB chemical shifts together. In this vein, two types of spectrum display modules are used, so-called “query” and “match” modules. This is very similar to the workflow previously developed in CcpNmr Version-2, but the user interface is now much simpler. In addition to spectrum displays, the backbone assignment workflow is assisted by three additional modules, i.e. the Sequence module, the Sequence Graph and the Backbone Assignment module. The complete setup of these different modules for backbone assignment in AnalysisAssign is shown in Fig. 6a.

To perform a backbone assignment, the user first selects the spectrum modules to use as match modules, using the adjustable parameters layout in the Backbone Assignment module (Fig. 3a). Once all the modules are set up, the process of finding matching strips is initiated by selecting an NmrResidue from the table in Backbone Assignment module. When an NmrResidue is selected, if it is an *i* − *1* NmrResidue (*see the data section*), the software examines all of the *i* NmrResidues and finds the closest matching CA^*i*^/CB^*i*^ pairs to the CB^*i*−*1*^/CA^*i*−*1*^ NmrAtoms of the NmrResidue. The closest matching NmrResidue is then connected to the selected NmrResidue forming a so-called “connected stretch.” A connected stretch is defined as an NmrChain wherein the NmrResidues are in a specific, sequential order. A connected stretch is displayed in the Sequence Graph, along with residue type predictions for each NmrResidue. In the GUI, connected stretches are formed by dragging and dropping strips, thus growing the connected stretch on either side depending on the ‘direction’ of the query.

The Backbone Assignment module has the built-in flexibility to perform assignment the *i* − *1* and *i* + *1* directions, depending on the NmrResidue selected. Moreover, the logic used to find matching NmrResidues is kept separate from the graphical code in the code base. This means that the introduction of a new assignment routine for matching NmrResidues can be easily introduced by changing just a few lines of code and the graphical manifestation of the workflow will remain unchanged.

Once a connected stretch contains more than three NmrResidues, possible assignment locations are assessed using a Bayesian-based chemical shift matching algorithm and highlighted in the Sequence Module. At this point the user can assign the NmrResidues to the sequence using drag-and-drop from the Sequence Graph to the Sequence Module.

The Sequence Graph can also be used to display any existing assignments within the project. If the different dimensions of a single peak in a spectrum are associated with assigned NmrAtoms, i.e. NmrAtoms that map to molecular atoms, this is displayed in the Sequence Graph as a line between the two atoms using the colour of the positive contours for that spectrum. The complete sequence of the protein can be shown in the Sequence Graph with all assignments displayed (Fig. 6b). This presents a simple way for a user to see what (molecular) atoms are assigned on the basis of which spectra and to spot any missing assignments very quickly. At present, this view is possible for backbone assignment, but the display of side chain assignments is under development for the subsequent version of AnalysisAssign. This mode can also be used to deassign i.e. disconnect the NmrResidue from the molecular residue.

**Fig. 7:**
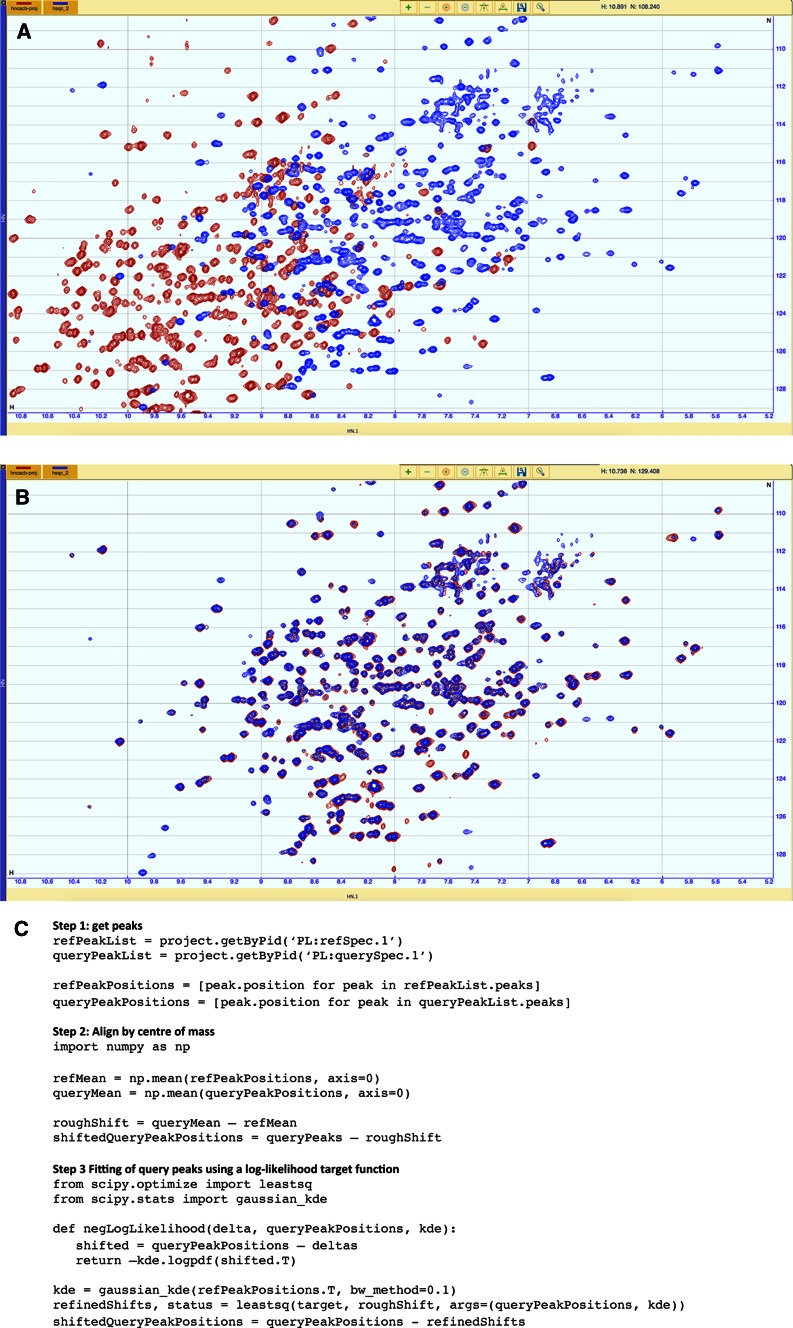
Correction of a mis-referenced HNCACB [^15^N, ^1^H] projection spectrum to overlay it with a [^15^N, ^1^H] HSQC spectrum, using a simple Python-based macro. **a** The HNCACB projection (*red*) and the HSQC spectrum (*blue*) do not overlay due to misreferencing of the HNCACB in the ^1^H and ^15^N dimension (1.5 ppm ^1^H, 4 ppm ^15^N, the differences in the reference points have been exaggerated for illustrative purposes). **b** After applying the automatically calculated referencing corrections, the HNCACB projection now overlays correctly with the HSQC spectrum. **c** A short (<20 *line*) python-based macro showing the three steps used to perform the referencing correction

AnalysisAssign has a Python-based macro language, allowing for the creation of macros using the Python programming language and the easy access to the AnalysisAssign data structures. Additionally, the functionality from any Python library can be used in such macros. As an example of this powerful functionality within AnalysisAssign, we examined a situation where two spectra of the same sample do not overlay, e.g. due to mis-referencing, altered sample conditions or different experimental setup. We wish to overlay these two spectra, even if the spectral content is not fully identical, i.e. there could be missing and/or additional intensities in either spectra (Fig. 7a).

The premise of the absence or presence of additional intensities precludes a simple peak comparison-based method. Instead, we opted for a two-stage approach in which a first coarse alignment is followed by a more fine-grained optimisation. We first obtain the peaks from both spectra, automatically picked if needed. The coarse initial alignment of the two spectra is derived from the difference of the centre of mass of the two peak sets, adjusting the query spectrum to the match spectrum accordingly. Subsequently, we perform a steepest descent minimisation using a log-likelihood target function based on a Gaussian kernel density estimator, as implemented in SciPy. The resulting alignment is very accurate (Fig. 7b) and a comparison with independent manual adjustments by four people yielded statistically identical results. Even though there is not a one-to-one correspondence between the two spectra, the alignment of the overlapping parts is excellent.

The macro to correct the referencing using this procedure is shown in Fig. 7c

Remarkably, it contains less than 20 lines of actual code and demonstrates the power and ease of extracting data from the project and then using external Python libraries to perform rather complex mathematics. A set of example macros covering various data access routines and different applications is provided as part of the distribution of AnalysisAssign.

## Concluding remarks

AnalysisAssign has been designed with great care and attention to comments from users of both existing CCPN software and other programs. As a result we have created a simple, functional and flexible software package, which is clean and straightforward to use, and suitable for all aspects of NMR data analysis, including liquid- and solid-state NMR data. The wrapper around the new data interface ensures facile interaction with underlying data and the graphical toolkit facilitates efficient creation of bespoke modules to interrogate and analyse data and calculations. We have also built a dedicated workflow for backbone assignment and one dedicated workflow for side chain assignment is currently under development. These workflows have been created in a modular manner so that users can modify and extend these, something that we would actively encourage. This is the first release of the CcpNmr version-3 suite and we are currently developing dedicated and closely related programs for NMR-based ligand screening, NMR-based metabolomics and NMR-based structure calculation.

## Additional information

Further information about the CCPN project as a whole can be found at http://www.ccpn.ac.uk. The AnalysisAssign package can be downloaded from http://www.ccpn.ac.uk/v3-software/downloads, all tutorial materials can be obtained from http://www.ccpn.ac.uk/v3-software/tutorials and the program-specific documentation can be obtained from http://www2.ccpn.ac.uk/docv3/build/html/index.html
